# Activity Analysis and Preliminary Inducer Screening of the Chicken *DAZL* Gene Promoter

**DOI:** 10.3390/ijms16036595

**Published:** 2015-03-23

**Authors:** Lei Zhang, Rui Zhu, Qisheng Zuo, Dong Li, Chao Lian, Beibei Tang, Tianrong Xiao, Yani Zhang, Bichun Li

**Affiliations:** College of Animal Science and Technology, Yangzhou University, Yangzhou 225009, Jiangsu, China; E-Mails: leizhang17@sina.com (L.Z.); rzhu1988@163.com (R.Z.); zqs081901427@gmail.com (Q.Z.); lidongyzu@hotmail.com (D.L.); lcyzdx@hotmail.com (C.L.); tangbeibei125@163.com (B.T.); xtryzdx2013@hotmail.com (T.X.)

**Keywords:** *DAZL* gene, core promoter, gene inducer screening, male germ cells

## Abstract

This study was aimed at identifying the active control area of chicken *DAZL* gene core promoter, to screen optimum inducers of the *DAZL* gene, thus to enhance the differentiation of embryonic stem cells into spermatogonial stem cells. Fragments of chicken *DAZL* gene promoter were cloned into fluorescent reporter plasmids and transfected into DF-1 cells. Then Dual-Luciferase^®^ Reporter Assay System was used to identify the activity of the *DAZL* gene under different inducers. Our studies showed that the *DAZL* core promoter region for the Suqin yellow chicken was −383 to −39 bp. The dual-luciferase^®^ reporter showed that all-trans retinoic acid (ATRA), a retinoic acid receptor alpha agonist (tamibarotene/Am80), or estradiol (E_2_) could significantly enhance *DAZL* transcription. The *in vitro* inductive culture of chicken ESCs demonstrated that, with ATRA treatment, *DAZL* transcription peaked at 6 days and then decreased slowly; whereas, *DAZL* transcription was continuous and peaked at 10 days with Am80 treatment. E_2_ treatment significantly increased *DAZL* expression after 8 days. All three treatments were associated with the appearance of male germ cell (MGC)-like cells on day 10. These results provide the optimum inducer screening of the *DAZL* gene and lay the foundation for further screening of compounds that can induce the differentiation of ESCs into MGCs *in vitro*.

## 1. Introduction

The autosomal *DAZL* gene is an important member of the Deleted in Azoospermia (DAZ) family (including *DAZ*, *DAZL* and *BOULE*), which encode RNA-binding proteins of the RNA recognition motif (RRM) type and are involved in translation of polysomal mRNA [1]. *DAZL*, which contains the conserved RRM repeat motif structural domain DAZ, was first discovered in the *Drosophila* testis [2]. Mouse *Dazl* expression starts during the early stage of germ cell development, persists into meiosis, and is expressed both in the adult testis and ovaries [3,4]. The RNA-binding protein encoded by *Dazl* is required for the completion of spermatocyte meiosis in the mouse [5]. Houston *et al.* [6] found that *Xenopus DAZL* was critically involved in primordial germ cell (PGC) development. Vogel *et al.* [7] determined that *Xenopus DAZL* could only substitute for the murine homologue with respect to the early functions in the establishment of the germ cells. Reynolds *et al.* [8] found that in *Dazl* knockout mice, meiotic prophase was arrested at the zygotene stage. Together these results indicate that *DAZL* has a conserved role in germ cell development, regulation of germ cell differentiation, and is one of the important regulatory factors in the process of gamete formation.

Currently, many research groups are focused on germ cell gene regulation and inductive differentiation of PGCs from embryonic stem cells (ESCs) *in vitro*. Yu [9] induced mouse ESCs to differentiate into sperm-like cells and oocyte-like cells by overexpression of *Dazl*. Haston [10] had shown that *Dazl* plays an important role in the process of differentiation from to germ cell *in vitro*. Using overexpression of *Dazl* Kee *et al.* [11] found that PGC formation could be promoted. Thus far, there have been a number of reports on mouse, human, and *Drosophila DAZL* and identification of a large number of inducers of its expression; however, the reports on the chicken ortholog are not comprehensive enough. Our team has been committed for decades to studying the inductive differentiation of chicken male germ cells (MGCs) *in vitro*. Unfortunately, the induction efficiency is low. To improve the induction efficiency, selecting the optimum inducers for chicken *DAZL* gene expression is crucial.

Chicken, as a classic model anima of developmental biology, displays a unique process of embryonic development. As an experimental model system, chickens provide sufficient material for the study of embryonic germ cells and have more permissive rules than mammalian models. The 5' flanking region of the *DAZL* gene promoter was cloned upstream of a fluorescent reporter and transfected into DF-1 cells. The Dual-Luciferase^®^ Reporter Assay System allowed quantification of the activity of the chicken *DAZL* promoter fragments and identification of the gene’s core promoter. The level of *DAZL* gene promoter activity was detected under different inducer treatments. Candidate optimal inducers were screened for their functional capacity *in vitro* to induce chicken ESCs to differentiate into MGCs, to provide the optimum inducer screening of the *DAZL* gene, and to lay the foundation for *in vitro* screening for inducers of ESC to MGC differentiation in the chicken.

## 2. Results and Discussion

### 2.1. The Qualitative Analysis of DAZL Gene Promoter Activity

**Figure 1:**
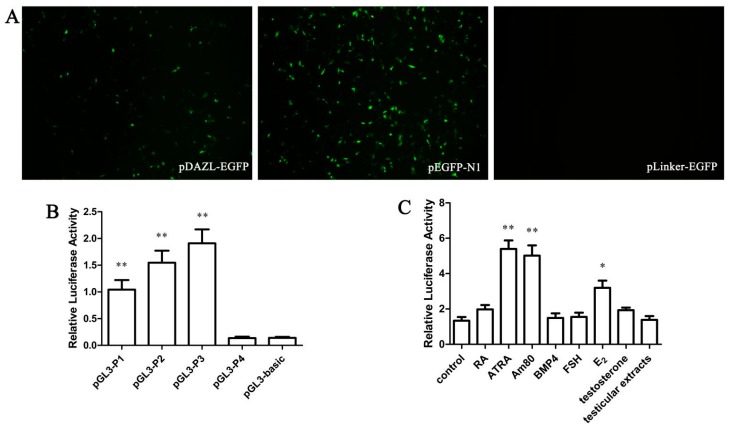
(**A**) The green fluorescent protein (GFP) detection of promoter activity of the chicken Deleted in Azoospermia (*DAZL*) long promoter fragment in DF-1 cells transfected with positive control *pDAZL-EGFP*, *pEGFP-N1*, and negative control *pLinker-EGFP* (40× magnification); (**B**) The activity of different promoter regions of the chicken *DAZL* gene in DF-1 cells; (**C**) The effect of different inducers on the activity of the chicken *DAZL* core gene promoter in mouse DF-1 cells. * represents *p* < 0.05, ** represents *p* < 0.01.

To test whether the fragment of −932 to −39 bp has promoter activity, *pDAZL-EGFP*, *pEGFP-N1*, and *pLinker-EGFP* plasmids were transfected into DF-1 cells (Figure 1A). The cells transfected with *pDAZL-EGFP* vector could express green fluorescent protein (GFP), but its fluorescence intensity was weaker than positive control plasmid *pEGFP-N1*, and *pLinker-EGFP* transfected cells had no GFP expression.

Four recombinant plasmids: *pGL-P1* (−932 to −39 bp), *pGL-P2* (−647 to −39 bp), *pGL-P3* (−383 to −39 bp), and *pGL-P4* (−186 to −39 bp) were used to transfect DF-1 cells with *pRL-SV40*. *pGL-basic* and *pRL-SV40* were used to transfect DF-1 cells as the negative control group. After 48 h of transfection, the activity of the four promoter segments was detected and different segments of the Suqin yellow chicken *DAZL* promoter had varying expression (Figure 1B). There was almost no promoter activity in the −186 to −36 bp fragment. The promoter activity in the −383 to −39 bp was the highest.

### 2.2. The Influence of Different Inducers on Chicken DAZL Gene Promoter Activity

According to the above result, *pGL3/334* GC-1 cells had the highest *DAZL* transcriptional activity. *pGL3/334* DF-1 cells were treated with RA (10^−5^ mol/L), ATRA (10^−5^ mol/L), Am80 (10^−6^ mol/L), BMP4 (40 ng/mL), FSH (0.1 IU/mL), E_2_ (1 μg/mL), testosterone (15 ng/mL), or testicular extract. After 48 h of induction, treatment with BMP4 or testicular extracts had no obvious effect on the activity of the *DAZL* gene promoter. RA, FSH, and testosterone increased the activity but not significantly. ATRA, Am80, and E_2_ significantly increased the activity of the *DAZL* gene promoter (Figure 1C).

### 2.3. In Vitro Induction Validation

**Figure 2:**
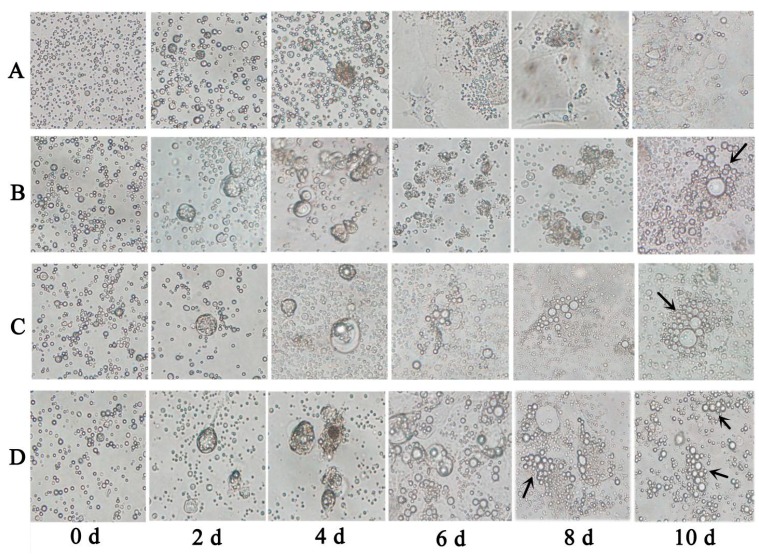
Differentiation of chicken embryonic stem cells (ESCs) induced by different *DAZL* inducers. (**A**) chicken ESCs with DMEM; (**B**) chicken ESCs with 10^−5^ mol/L l-trans retinoic acid (ATRA) induction; (**C**) chicken ESCs with 10^−6^ mol/L Am800 induction; (**D**) chicken ESCs with 1 μg/mL E_2_ induction. Arrows represent spermatogonial stem cell-like (SSC-like) cells. (400× magnification).

Based on their induction of *DAZL* transcription ATRA, Am80, and E_2_ were screened as the optimal inducers of the *DAZL* gene. Morphological changes were induced in chicken ESCs *in vitro* by ATRA, AM80, and E_2_. qRT-PCR and immunohistochemistry were used to detect the kinetics of *DAZL* gene expression during differentiation. The results showed that cell morphology gradually changed under a different culture system (Figure 2). Under basic medium, control cells grew slowly. A few cell colonies appeared at 2 days, and the cells differentiated at 6 days. With ATRA or Am80 treatment, embryonic bodies gradually formed and increased at 2 days, began to collapse at 6 days, and MGC-like cells were observed at 10 days. With E_2_ treatment, embryonic bodies gradually formed and enlarged at 2 days, began to collapse at 6 days, MGC-like cells were observed at 8 days, and the number increased at 10 days.

**Figure 3:**
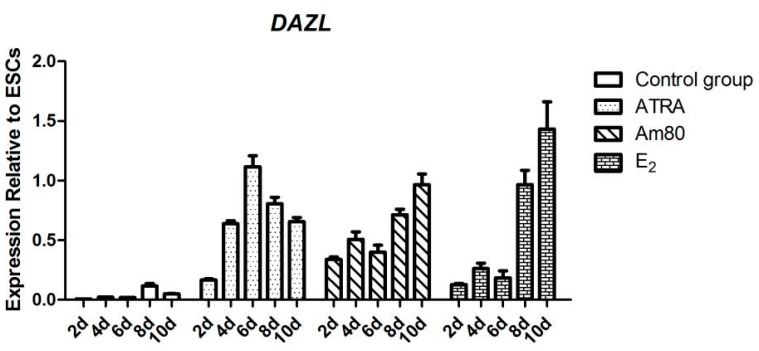
*DAZL* mRNA expression under ATRA, Am80, and E_2_ induction compared with control chicken ESCs. The results are presented as a mean ± SEM of three duplicate runs. Error bars in charts represent the corresponding standard deviations.

**Figure 4:**
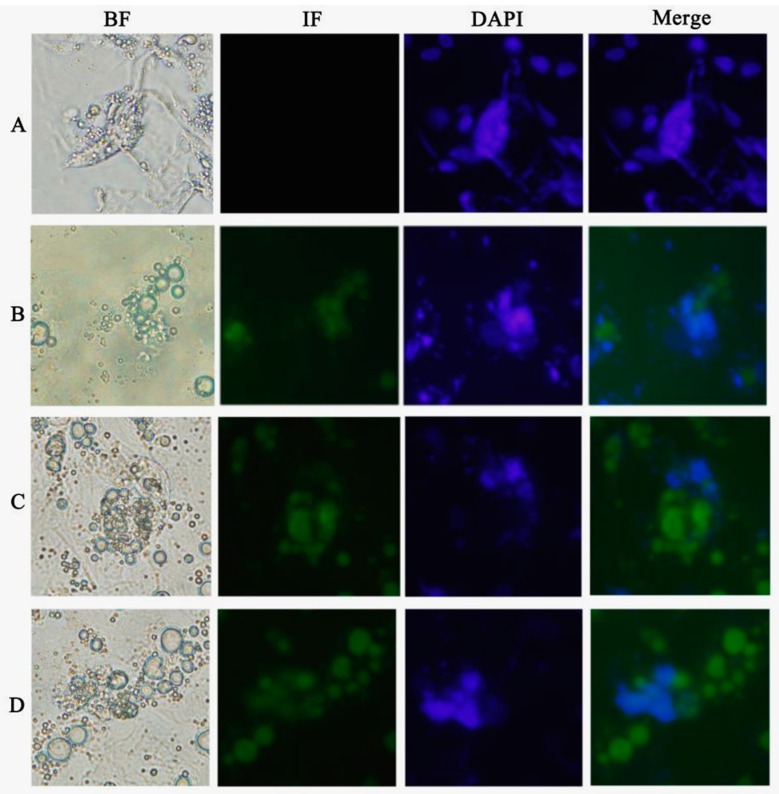
Immunohistochemical detection of DAZL protein expression in chicken ESCs treated with various inducers of germ cell differentiation. (**A**) chicken ESCs with DMEM; (**B**) chicken ESCs with 10^−5^ mol/L ATRA induction; (**C**) chicken ESCs with 10^−6^ mol/L Am80 induction; (**D**) chicken ESCs with 1 μg/mL E_2_ induction. (400× magnification).

qRT-PCR results showed that, compared with the control group, *DAZL* gene expression achieved the highest level at 6 days of ATRA treatment, and then showed a trend of slow decline. *DAZL* gene showed continuous expression after Am80 treatment, achieving the highest level at 10 days. *DAZL* gene expression was significantly increased at 8 days of E_2_ treatment (Figure 3). Immunohistochemistry also showed that with ATRA, Am80, or E_2_ treatment, the cells expressed DAZL protein (green), which was absent in the control cells (Figure 4).

## 3. Experimental Section

### 3.1. Materials and Reagents

Procedures involving animals and their care conformed to the U.S. National Institute of Health guidelines (NIH Pub. No. 85-23, revised 1996) and were approved by the laboratory-animal management and experimental-animal ethics committee of Yanzhou University.

Fertilized eggs of Suqin yellow chickens (*Gallus gallus domesticus*) were purchased from the Chinese Academy of Agricultural Sciences Experimental Poultry Farm (Yangzhou, China). ESCs were collected from stage X embryos as described previously [12].

The *pEGFP-N1* vector is maintained by our laboratory. *E. coli* DH5α competent cells, gel extraction kits, and miniprep kits were purchased from Tiangen Biotech (Beijing, China). DL5000 DNA Marker Prime, STAR Max DNA Polymerase, T4 DNA ligase, and restriction endonucleases were purchased from Takara (Shiga, Japan). Expression vectors *pGL3.0-Basic*, *pRL-SV40*, and the dual-luciferase assay kit, Dual-Luciferase Reporter Assay System, were purchased from Promega Corporation (Madison, WI, USA). LipofectamineTM2000 was purchased from Invitrogen Life Technologies (Carlsbad, CA, USA). Retinoic acid (RA), all-trans retinoic acid (ATRA), tamibarotene (Am80), estrogen (E_2_), and testosterone were purchased from SIGMA-Aldrich (St. Louis, MO, USA). Mouse bone morphogenetic protein 4 (BMP4) and follicle stimulating hormone (FSH) were purchased from ProSpec-Tany TechnoGene Ltd. (Rehovot, Israel). The mouse spermatogonial cell line (GC-1) and the chicken embryo fibroblast cell line (DF-1) were purchased from ATCC (Manassas, VA, USA). The remaining reagents were imported or domestic analytical grade. Primer synthesis and sequencing were conducted by Invitrogen.

### 3.2. Extraction of Genomic DNA

Genomic DNA was extracted from chicken peripheral blood samples using a Puregene DNA isolation kit (Gentra, Minneapolis, MN, USA) according to the manufacturer’s instructions.

### 3.3. Genomic Clone

Four fragments of the chicken *DAZL* gene promoter were amplified by polymerase chain reaction (PCR), forward primers (positions relative to the transcription start site, corresponding to the genomic positions chr2:34953772-34954681 for the longest amplicon) were: pEGFP-P0 (−932 to −39 bp): 5'-CGCATTAATTATGCACTCCAGTTGATCAGTTTAA-3', pGL-P1 (−932 to −39 bp): 5'-GGGGTACCTATGCACTCCAGTTGATCAGTTTAA-3', pGL-P2 (−647 to −39 bp): 5'-GGGTACCCCAGCCTGCTCCAGAGTATCCA-3', pGL-P3 (−383 to −39 bp): 5'-GGGGTACCCACACATCGCCCTTCGTCTT-3', pGL-P4 (−186 to −39 bp): 5'-GGGGTACCAAGAACTGCCTCTTTCGCAC-3', and common reverse primer: 5'-CCCTCGAGCAAACGAGGCCTTCAAGACAA-3'.

The PCR consisted of 35 cycles of gene amplification as follows: 98 °C for 10 s followed by 35 cycles composed of a denaturation step at 95 °C for 30 s, 56–62 °C for 30 s, and an elongation step at 72 °C for 40 s. The PCR products were resolved by electrophoresis on 1% agarose gels, and the appropriate band was excised and purified for the subsequent steps of cloning or sequence determination. Sequence analysis was performed with an automatic sequencer (ABI 377, Applied Biosystems/PE/Life Technologies, Grand Island, NY, USA).

### 3.4. Vector Construction

The *DAZL* gene 5' flanking area −932 to −39 bp was inserted into the *pEGFP-N1* vector between *Ase*I and *Xho*I restriction enzyme cutting sites and named *pDAZL-EGFP*. A negative control carrier was also built as *pLinker-EGFP*. Four fragments of the *DAZL* gene promoter region cloned previously were inserted into the dual-luciferase reporter gene carrier *pGL3-Basic*. All vectors were verified by *Ase*I and *Xho*I restriction enzyme digestion and sequence analysis.

### 3.5. Qualitative Analysis of DAZL Gene Promoter

DF-1/GC-1 cells were inoculated into 12 wells, approximately 5 × 10^5^ cells per well. According the manufacturer’s instructions for LipofectamineTM2000, *pEGFP-N1/893* was used for transfection of DF-1/GC-1 cells, and a positive (*pEGFP-N1* transfected cells) and negative control group (*pEGFP-N1-CMV* transfected cells) were created. The results for each group represent three replications. After 48 h of transfection, fluorescence was observed under a Nikon Eclipse microscopy (Nikon, Tokyo, Japan).

### 3.6. Cell Transfection

DF-1/GC-1 cells were inoculated into 24 wells, approximately 2.5 × 10^5^ cells per well. According to the manufacturer’s instructions for LipofectamineTM2000, all recombinant plasmids and a reference plasmid *pRL-SV40* (35:1) were transfected into DF-1/GC-1 cells. DF-1/GC-1 cells transfected with *pGL3-basic* and *pRL-SV40* were selected as a negative group. The results from each group represent three replications. After 48 h, the Dual-Luciferase^®^ Reporter Assay was performed.

After 6 h of transfection, cells were treated with RA (10^−5^ mol/L), ATRA (10^−5^ mol/L), Am80 (10^−6^ mol/L), BMP4 (40 ng/mL), FSH (0.1 IU/mL), E_2_ (1 μg/mL), testosterone (15 ng/mL), or testicular extract. The negative group was treated with the same volume of phosphate-buffered saline. After treatment for 48 h, cells were collected. The results of each group represent three replications.

### 3.7. Dual-Luciferase Activity Detection

Cells were collected in 70 μL PBS and the suspension was transferred to a 96-well plate. A Dual-Luciferase^®^ Reporter Assay was performed to detect the activity of the *DAZL* gene promoter constructs. The relative luciferase activity representing the promoter activity is reported as the ratio of firefly luciferase value/renilla luciferase.

### 3.8. In Vitro Induction Experiment

Chicken ESCs were inoculated into 24 wells, approximately 2 × 10^5^ cells per well. ATRA (10^−5^ mol/L), Am80 (10^−6^ mol/L), and E_2_ (1 μg/mL) were used to induce chicken ESC differentiation *in vitro*. The cells were collected every two days after inducer treatment. The results of each group consisted of three replications.

### 3.9. Quantitative Real-Time Reverse Transcriptase PCR (qRT-PCR)

Total RNA was extracted using the RNeasy kit (Qiagen, Suzhou, China). Reverse transcription into cDNA was performed by Reverse Transcription System (Qiagen) to serve as a template for qRT-PCR. qRT-PCR of the cDNA was performed according to the instructions provided in the fluorescence quantitative PCR kit (Qiagen), using SYBR as the fluorescent reagent and 7500 System fluorescence quantitative instrument (Applied Biosystems, Foster City, CA, USA). Each group had three replications. The data were analyzed in Microsoft Excel using the 2^−ΔΔ*C*t^ relative quantitative method.

## 4. Discussion

*DAZL* is one of the DAZ family members encoding an RNA-binding protein, which is also an indispensable control factor during the animal gametogenesis process. To explore the role of the *DAZL* gene in the specific regulatory mechanism of male reproductive cells, different lengths of the chicken *DAZL* gene promoter attached to a fluorescent reporter were constructed, and were transfected into DF-1. A Dual-Luciferase^®^ Reporter Assay was performed, showing that the *pGL-P3* promoter has the highest activity of the *DAZL* gene promoter fragments in DF-1 cells. This indicates that there are important regulatory elements in the −382 to −39 bp region of the chicken *DAZL* gene promoter.

Zhuo *et al.* [13] found that with ectopic expression of the *Dazl* gene, both motile-tailed sperm and oocytes could be induced from mouse ESCs. Several other groups [14,15,16,17] also verified that *Dazl* was a master gene controlling germ cell differentiation. However, *DAZL* gene research is not comprehensive in chickens, and there is doubt whether specific inducers of the mouse *Dazl* gene could be used for directional induction by the chicken ortholog *in vitro*. To evaluate the conservation of *DAZL* gene function, combining previous research results [18,19,20], RA, ATRA, Am80, BMP4, FSH, E_2_, testosterone, and testicular extracts evaluated for induction of *pGL3/334* GC-1 cells.

BMP4 is a multifunctional cytokine, which can induce ESCs to differentiate into MGCs *in vitro*. Shi *et al.* [20] found that the expression of *Dazl*, *Stra8*, *Integrin α6*, and *c-kit* was enhanced with every 2 days of BMP4 treatment in chicken. In our study, BMP4 treatment did not enhance the activity of the core chicken *DAZL* gene promoter. Considering the short treatment time of BMP4 in our study (48 h), the lack of an effect might be because BMP4 does not directly affect the chicken *DAZL* gene, but indirectly causes the expression of *DAZL* mRNA through interaction with multiple genes. Neither testosterone nor testicular extract had an effect on the chicken *DAZL* gene promoter, which is consistent with the results by Sun and colleagues [12]. Pan *et al.* [19] found that E_2_ and FSH could induce mouse ESCs to differentiate into MGCs with high-level expression of mouse *Dazl* mRNA on day 7. Our study is consistent with their earlier findings. Additionally E_2_ had a more significant effect than FSH in our hands. Retinoic acids, including ATRA and RA, are fat-soluble small molecule metabolites of vitamin A. RA plays an important role in biological development and normal physiological conditions. The compound can mediate cell differentiation, proliferation, apoptosis, and regulate immune function. Kerkis and colleagues found that, 10^−5^ mol/L of RA could induce mouse ESCs to differentiate into MGCs positive for *Dazl* gene expression. The single factor induction by ATRA and RA in our study showed that both can enhance the expression of the chicken *DAZL* gene. Am80 is a new synthetic retinoic acid analogue and specific receptor agonist of RARα. Am80 has a higher induced efficiency than ATRA or RA.

Combining with the prediction of regulatory elements binding sites of chicken *DAZL* gene (Figure S3), a RAR-beta (RARβ) binding site presented in the gene’s core promotor region. This indicated that ATRA and RA could probably promoting chicken *DAZL* gene transcription by directly binding this interval. The absence of ER (estrogen receptor) and RARalpha (RARα) binding site indicating that E_2_ or Am80 might play its role through other bound transcription factors or by signaling. We also found two AR (androgen receptor) binding sites in this region, ATRA, Am80 or E_2_ could also directly binding this interval to paly the role in chicken *DAZL* gene transcriptional regulation. We are investigating further about the specific molecular mechanism of chicken *DAZL* gene inducer. 

To further validate whether the inducers we screened from chicken DF-1 cells could be applied to other species, we also used mouse GC-1 cells for the same test (Figure S1). The results showed that ATRA, Am80, and E_2_ could also significantly increased the activity of the *DAZL* gene promoter in GC-1 cells (Figure S2C). ATRA, Am80, and E_2_ can significantly enhance the activity of the chicken *DAZL* gene promoter and expression of the encoded DAZL protein. The formation of MGC-like cells was also observed during the induction, indicating that these factors can regulate the minimal chicken *DAZL* promoter similar to its mammalian counterparts.

## 5. Conclusions

In this paper, we identified the core promoter region of the *DAZL* gene for the Suqin yellow chicken, and compared the induction efficiency of different inducers. ATRA, Am80, and E_2_ were screened as optimal inducers of the chicken *DAZL* gene, and *in vitro* induction validation was performed. This study provides a starting point to design the optimal culture system to induce chicken ESCs to differentiate into MGCs *in vitro*, in part, by significantly raising *DAZL* gene expression.

## Supplementary Materials

Supplementary materials can be found at http://www.mdpi.com/1422-0067/16/03/6595/s1.
