# Supplementary material for: Activity Analysis and Preliminary Inducer Screening of the Chicken DAZL Gene Promoter
Source: Int J Mol Sci. 2015 Mar 23;16(3):6595–605. doi: 10.3390/ijms16036595 (PMC4394550; doi:10.3390/ijms16036595)
Supplement: Supplementary file 1 [file ijms-16-06595-s001.pdf]

Supplementary Information

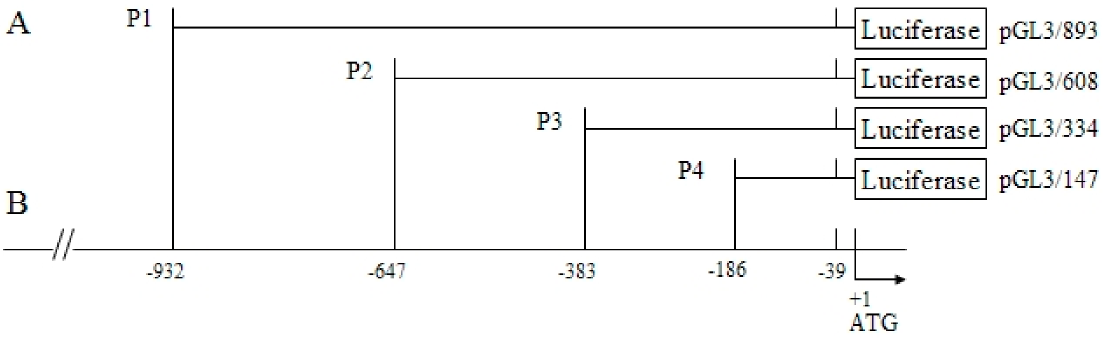

Figure S1. The primer design scheme for deletional analysis of the chicken *DAZL* gene promoter region.

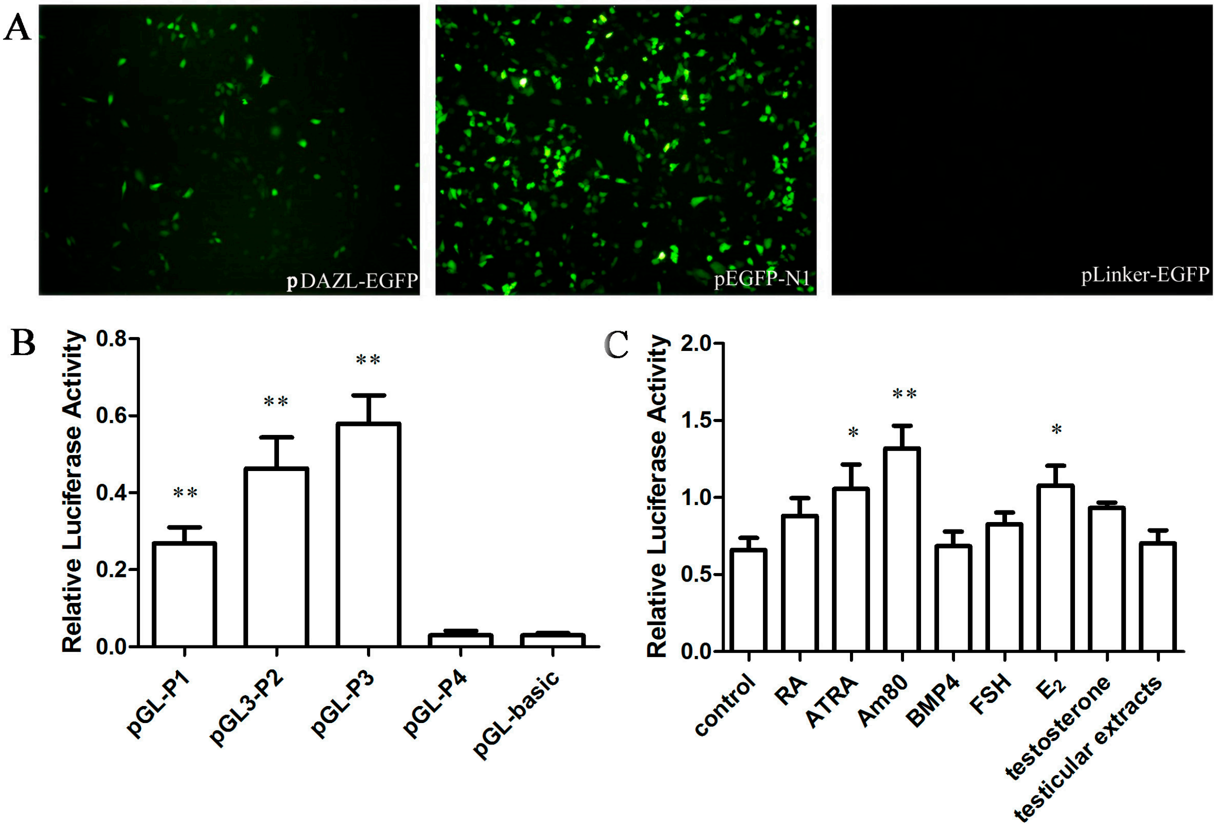

Figure S2. (**A**) The GFP detection of promoter activity of the chicken *DAZL* long promoter fragment in GC-1 cells transfected with positive control *pDAZL-EGFP*, *pEGFP-N1*, and negative control *pLinker-EGFP* (40× magnification); (**B**) The activity of different promoter regions of the chicken *DAZL* gene in GC-1 cells; (**C**) The effect of different inducers on the activity of the chicken *DAZL* core gene promoter in mouse GC-1 cells. * represents $p < 0.05$, ** represents $p < 0.01$.

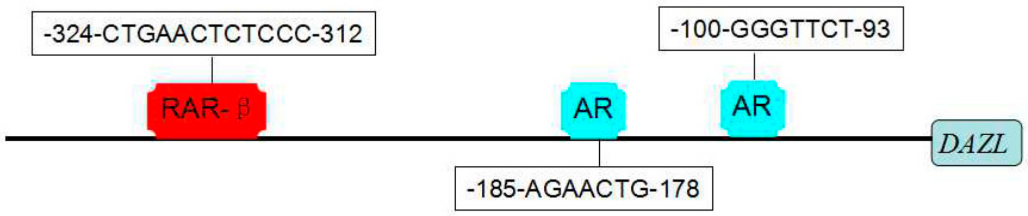

Figure S3. Prediction of regulatory elements binding sites at −383~−36 bp of chicken *DAZL* gene.
